# Vaccination in Immune-Mediated Intestinal Diseases: Efficacy, Safety, and Future Directions

**DOI:** 10.3390/vaccines14050426

**Published:** 2026-05-09

**Authors:** Adi Lahat, Kassem Sharif

**Affiliations:** 1 Department of Gastroenterology, Samson Assuta Ashdod Medical Center, Faculty of Medicine, Ben Gurion University of the Negev, Be’er Sheva 7747629, Israel; 2 Advanced Endoscopy Unit, Beth Israel Deaconess Medical Centre, Faculty of Medicine, Harvard Medical School, Boston, MA 02215, USA; kassemsharif@gmail.com

## 1. Introduction

Vaccination remains one of the most effective public health interventions for preventing infectious diseases, reducing morbidity, mortality, and healthcare burden worldwide. In patients with immune-mediated intestinal diseases (IMIDs), particularly inflammatory bowel diseases (IBD) such as Crohn’s disease and ulcerative colitis, vaccination has special importance because these patients are at increased risk of vaccine-preventable infections due to chronic immune dysregulation, malnutrition, surgery, comorbidities, and widespread use of immunosuppressive therapies including corticosteroids, thiopurines, methotrexate, anti-TNF agents, anti-integrins, anti-IL12/23 biologics, JAK inhibitors, and combination regimens [1,2]. Preventing infections in this population is therefore an essential component of comprehensive disease management.

Over the last decade, substantial progress has been made in defining vaccine strategies for patients with IMIDs. Major international societies now recommend systematic assessment of immunization status at diagnosis and proactive administration of routine inactivated vaccines, including influenza, pneumococcal, hepatitis B, human papillomavirus, COVID-19, and recombinant herpes zoster vaccines when appropriate [3,4]. These recommendations reflect accumulating evidence that most inactivated vaccines are safe in IBD populations and do not increase disease activity. However, vaccine uptake remains suboptimal in many cohorts because of fragmented care pathways, uncertainty regarding responsibility between specialists and primary care providers, and persistent patient hesitancy [5].

A major challenge in this field is vaccine immunogenicity under immunosuppressive treatment. Several studies have shown attenuated serologic responses to influenza, pneumococcal, hepatitis B, and SARS-CoV-2 vaccines among patients receiving anti-TNF therapy, systemic corticosteroids, JAK inhibitors, or combination immunosuppression [6,7,8]. Conversely, newer gut-selective agents such as vedolizumab may have less systemic impact on vaccine responsiveness. These observations have highlighted the need for optimized vaccine schedules, booster strategies, post-vaccination antibody monitoring in selected populations, and individualized timing of vaccination before treatment escalation whenever feasible.

The COVID-19 pandemic markedly accelerated research in vaccination among patients with immune-mediated intestinal diseases. Large multicenter registries demonstrated that SARS-CoV-2 vaccines were generally safe, highly effective against severe disease, and not associated with clinically meaningful increases in IBD flares in most patients [4,9]. At the same time, reduced humoral durability in anti-TNF-treated patients and improved responses after booster doses emphasized the importance of tailored vaccine programs in immunocompromised hosts [10].

Despite these advances, important knowledge gaps remain. Real-world effectiveness data for newer vaccines, durability of protection across drug classes, correlates of cellular immunity, vaccine responses in pediatric and elderly IBD populations, optimal management of live vaccines in selected settings, and implementation strategies to improve uptake are still insufficiently defined. This Special Issue, “Vaccine Efficacy and Safety in Patients with Immune-Mediated Intestinal Diseases,” addresses these unmet needs by bringing together contemporary evidence on immunogenicity, safety, clinical outcomes, and preventive strategies in this growing and increasingly complex patient population. Collectively, the contributions reinforce that vaccination should be viewed not as an adjunct but as a core pillar of modern care for patients with immune-mediated intestinal disease.

## 2. Overview of the Contributions in This Special Issue

This Special Issue, “Vaccine Efficacy and Safety in Patients with Immune-Mediated Intestinal Diseases,” brings together timely original investigations and comprehensive reviews that address key unanswered questions regarding vaccine responsiveness, durability, and safety in patients with chronic immune-mediated disorders. Collectively, the manuscripts included highlight that although vaccination remains broadly safe and clinically beneficial in these populations, vaccine-induced immunity may be quantitatively and qualitatively modified by the underlying disease state, associated immune dysfunction, and immunosuppressive treatment regimens.

A central theme of the issue is vaccination in inflammatory bowel disease (IBD), where biologic and small-molecule therapies have transformed disease outcomes but also introduced new complexity in infection prevention. Vollenberg et al. demonstrated that even after a third SARS-CoV-2 mRNA vaccine dose, immunosuppressed IBD patients exhibited reduced humoral responses compared with healthy controls, with the most pronounced impairment observed in patients receiving anti-TNF therapy [11]. These findings reinforce prior concerns that anti-TNF treatment may blunt vaccine immunogenicity and may justify closer booster surveillance in selected patients.

Building upon this concept, Woelfel et al. evaluated monovalent XBB.1.5-adapted COVID-19 mRNA vaccines in IBD patients and showed significant enhancement of systemic neutralizing responses against contemporary Omicron-related variants, including EG.5.1 and BA.2.86 [12]. However, anti-TNF-treated individuals again showed lower neutralization capacity, emphasizing that variant-updated boosters improve protection but do not fully eliminate treatment-associated immune attenuation. In a subsequent companion study, the same group further explored mucosal immunity and demonstrated that although XBB.1.5 vaccination induced salivary IgG responses, mucosal IgA responses remained inadequate in IBD patients [13]. Given the pivotal role of secretory IgA in preventing infection at viral entry sites, these data provide an important mechanistic explanation for breakthrough infections despite preserved systemic immunity and strongly support future development of mucosal vaccine platforms for immunocompromised hosts.

Beyond IBD, this Special Issue broadens the discussion to other immune-mediated intestinal disorders through an extensive review of vaccination in celiac disease by Scarmozzino et al. [14]. The authors summarize evidence showing that most routine vaccines are effective and safe in celiac disease, while highlighting specific clinical considerations such as hyposplenism, selective IgA deficiency, altered mucosal barrier function, and reduced responsiveness to hepatitis B vaccination. Their review importantly reframes celiac disease as a condition in which personalized vaccine planning may be appropriate, particularly for adults with splenic dysfunction or additional autoimmune comorbidities.

Although outside the strict intestinal disease spectrum, the prospective cohort study by Alexeeva et al. adds valuable comparative insight into vaccination under pediatric immune-mediated conditions [15]. In children with juvenile idiopathic arthritis receiving immunomodulatory therapy, simultaneous pneumococcal and Haemophilus influenzae type b vaccination was immunogenic, clinically beneficial, and not associated with meaningful disease flares. These findings are highly relevant to gastroenterologists managing pediatric IBD, where concerns regarding flare induction continue to contribute to vaccine hesitancy.

Taken together, the studies in this Special Issue converge on several major messages. First, non-live vaccines remain safe across a broad range of immune-mediated diseases. Second, immunogenicity cannot be assumed to be equivalent to that of the general population, particularly in patients treated with anti-TNF agents or combination immunosuppression. Third, conventional serum antibody measurements alone may be insufficient, as mucosal immune protection may remain suboptimal. Finally, vaccine delivery in immune-mediated diseases should evolve toward precision prevention strategies that integrate disease phenotype, age, therapy class, prior infection history, and emerging correlates of immune protection.

## 3. Conclusions

This Special Issue demonstrates that vaccination in patients with immune-mediated intestinal diseases should be regarded as a central component of disease management rather than a secondary preventive measure. Across inflammatory bowel disease, celiac disease, and related immune-mediated conditions, the studies included herein consistently show that non-live vaccines are generally safe, well tolerated, and clinically beneficial, without meaningful evidence of disease destabilization or excess adverse events. At the same time, they highlight that vaccine responses in these populations are not uniform and may be substantially influenced by immune dysregulation, age, comorbidities, and particularly immunosuppressive therapies such as anti-TNF agents.

A major contribution of this Special Issue is the recognition that vaccine efficacy extends beyond conventional serum antibody titers. While updated COVID-19 vaccines improve systemic neutralizing immunity, persistent deficiencies in mucosal immune responses may help explain continued susceptibility to breakthrough infections, especially in patients with IBD. These findings support a broader and more sophisticated framework for evaluating vaccine protection that incorporates humoral, cellular, and mucosal immunity rather than relying solely on circulating antibody levels.

Collectively, these contributions emphasize the need for a precision-vaccination approach tailored to the individual patient. Future strategies should focus on optimizing vaccine timing relative to immunosuppressive therapy, defining booster schedules according to treatment class and immune response durability, improving vaccine uptake through integrated gastroenterology-led preventive care pathways, and developing next-generation mucosal or variant-adapted platforms for vulnerable populations. As the therapeutic landscape of immune-mediated intestinal diseases continues to evolve, proactive and personalized vaccination strategies will remain essential to reducing infectious morbidity and improving long-term patient outcomes.
